# Cluster-Based Strategy for Maximizing the Sum-Rate of a Distributed Reconfigurable Intelligent Surface (RIS)-Assisted Coordinated Multi-Point Non-Orthogonal Multiple-Access (CoMP-NOMA) System

**DOI:** 10.3390/s24113644

**Published:** 2024-06-04

**Authors:** Qingqing Yang, Qiuhua Zhang, Yi Peng

**Affiliations:** Faculty of Information Engineering and Automation, Kunming University of Science and Technology, Kunming 650500, China; 20090119@kust.edu.cn (Q.Y.); 18377335701@163.com (Q.Z.)

**Keywords:** RIS, CoMP-NOMA, alternating optimization, user cluster, phase-shift design

## Abstract

This article proposes a distributed intelligent Coordinated Multi-Point Non-Orthogonal Multiple-Access (CoMP-NOMA) collaborative transmission model with the assistance of reconfigurable intelligent surfaces (RISs) to address the issues of poor communication quality, low fairness, and high system power consumption for edge users in multi-cellular networks. By analyzing the interaction mechanisms and influencing factors among RIS signal enhancement, NOMA user scheduling, and multi-point collaborative transmission, the model establishes RIS-enhanced edge user grouping and coordinates NOMA user clusters based on this. In the multi-cell RIS-assisted JT-CoMP NOMA downlink transmission, joint optimization of the power allocation (PA), user clustering (UC), and RIS phase-shift matrix design (PS) poses a challenging Mixed-Integer Non-Linear Programming (MINLP) problem. The original problem is decomposed by optimizing the formulas into joint sub-problems of PA, UC, and PA and PS, and solved using an alternating optimization approach. Simulation results demonstrate that the proposed scheme effectively reduces the system’s power consumption while significantly improving the system’s throughput and rates.

## 1. Introduction

With the widespread application of fifth-generation mobile communication, the surge in data traffic poses increasingly higher requirements for wireless communication, including higher data rates, ultra-high communication reliability, and low transmission latency. Consequently, 5G networks will face higher power consumption and deployment costs [1]. RISs, one of the innovations in next-generation communication technologies, utilize intelligent surfaces to modify the propagation characteristics of electromagnetic waves, optimizing signal transmission and reception to enhance spectrum efficiency with lower power consumption and hardware costs [2]. Introducing RISs into communication environments can achieve better signal quality and coverage enhancement, striking a balance between power efficiency and communication performance.

In traditional cellular networks, NOMA technology is often utilized as one of the technical means to reduce system power consumption due to its ability to achieve high spectrum efficiency and support massive connectivity [3,4]. NOMA allows multiple users to transmit data simultaneously at the same time and with the same frequency resources, thereby improving the spectrum efficiency through non-orthogonal signal transmission between users. This enables support for more users within limited spectrum resources, reducing the need for additional spectra and thus aiding in lowering system power consumption [5]. Recently, researchers have studied RIS-assisted multi-user NOMA systems, developing related network transmission strategies and conducting performance analyses. The main methods typically fall into three types: optimization methods based on beamformer strategies, optimization methods based on cluster strategies, and optimization methods based on coordinated cluster strategies.

1. Optimization Methods Based on Beamformer Strategies: Each user employs an independent beamformer for service provision in the specific framework based on beamformers. The research in this area primarily focuses on the design of passive and active beamformers and NOMA user power allocation. For a dual-user RIS-assisted Multiple-Input Single-Output (MISO) NOMA downlink, Zheng et al. [6] improved energy efficiency by alternately optimizing passive and active beamformers. They developed active beamforming matrices at the base station and passive beamforming matrices at the RIS using continuous convex approximation and semi-definite relaxation, respectively. Zeng et al. [7] developed a new algorithm using a sequential rank-one relaxation method in RIS-assisted NOMA systems to find a locally optimal rank-one solution. Peng et al. [8] studied the alternating optimization of beamforming in the downlink RIS-NOMA system, proposing a method that combines fractional programming with alternating optimization of main and passive beamforming using continuous convex approximation. Yang et al. [9] researched an uplink NOMA system assisted by active RISs and rate optimization. They combined active RISs with NOMA technology for the first time, introducing constraints on the amplitude and amplification power of active RISs to establish a problem of maximizing rates. Björnson et al. [10] proposed a new resource allocation algorithm, a low-complexity decoding order optimization algorithm, and a joint optimization algorithm to, respectively, address channel allocation, decoding order, power allocation, and reflection coefficient design issues.
2. Optimization Methods Based on Cluster Strategies: Faced with large-scale user communications, a clustering strategy can improve user fairness and reduce system complexity. The key idea is to divide users into different clusters, with each cluster using a common beamformer to serve the users within that cluster [11]. Researchers have combined RIS deployment designs to study both centralized RIS and distributed RIS-assisted NOMA systems based on user clusters. Zuo et al. [12], targeting mmWave communication systems, adopted a centralized cluster NOMA strategy based on RISs. Data verification showed that the RIS-based centralized cluster NOMA scheme outperformed benchmark schemes based on ZF and OMA. Ding et al. [13] deployed multiple distributed RISs to serve edge users of a cell using a clustering strategy. They paired an edge user with a central cell user to form a NOMA cluster. Simulation data indicated that the NOMA strategy enhanced by distributed RISs could increase the maximum number of successfully served users and the coverage range of the BS. Xiu et al. [14] jointly optimized the beamforming vector at the BS and the RIS element matrix in a multi-cluster RIS-assisted MISO-NOMA communication network to minimize transmission power. Mu et al. [15] addressed the optimization problem of user clustering, power, and resource allocation in the downlink of a NOMA system, maximizing the minimum successful probability of user service.
3. Optimization Methods Based on Coordinated Cluster Strategies: In multi-cell, multi-user communication systems, due to the use of the same frequency for networking, edge users of cells will experience severe inter-cell interference [16,17]. To solve the technical challenge of communication transmission, CoMP allows multiple base stations to simultaneously transmit to specific terminals by coordinating the transmission and reception of multiple base stations and terminals, thus forming multi-point joint transmission [18,19,20]. The use of multi-cell MIMO technology provides the conditions for using CoMP technology to solve the problem of inter-cell interference. By utilizing the characteristics of the spatial channel to transmit signals, the introduction of CoMP technology improves the performance of edge users. Studies have shown that integrating RISs into multi-cell CoMP systems can further increase the transmission rate of edge users. Mohamed Elhattab et al. [21,22] studied the integration of RISs with downlink NOMA in a JT-CoMP-assisted dual-cell network. The RIS was deployed at the edge of two adjacent cells. By jointly optimizing the power allocation coefficients at the base station, employing user-clustering strategies, and using the phase-shift matrix of the RIS, they achieved maximization of the network and rate. Sun et al. [23] derived the outage probability and throughput of an uplink CoMP NOMA network using stochastic geometry, wherein a BS with a single antenna in the cell only serves one CoMP user. Wang et al. [24] investigated the problem of maximizing the sum-rate in a downlink RIS-assisted CoMP network with Device-to-Device (D2D) communication, further addressing this issue using semi-definite relaxation and penalty methods. As shown in Table 1, a comparison has been made between the work presented in this paper and the existing work published in recent years.

Therefore, in scenarios where there is deep overlapping coverage among multiple cells, it is essential to analyze and deeply explore the core foundational issues of RIS-assisted multi-cell, multi-antenna, NOMA-coordinated multi-point transmission. By modeling the interaction mechanism among RIS, JT-CoMP, and NOMA technology, and conducting in-depth analyses and attempts to resolve issues such as user scheduling, clustering, and power allocation, a novel optimization method for distributed RIS-assisted JT-CoMP NOMA network transmission is established. This can provide excellent technical support for analyzing the performance enhancement mechanism of introducing RISs into multi-cell CoMP NOMA networks.

Based on the background provided above, the main contributions of this paper are as follows:

The technology mentioned in the literature [12,15,18,22,25,28] is mainly applied to systems with single-RIS-assisted communication. However, in actual communication scenarios, with a large number of users, the resource utilization rate of single-RIS-assisted communication is relatively low. To better meet user demands and enhance system availability and flexibility, this paper proposes a distributed RIS-assisted CoMP-NOMA downlink transmission system model. It investigates the problems of minimizing power consumption while ensuring the lowest QoS (Quality of Service) for users and maximizing the transmission rate in a distributed RIS-assisted CoMP-NOMA system. By jointly optimizing inter-group power allocation, intra-group power allocation, user grouping, and phase-shift optimization, the system aims to reduce power consumption and improve system performance and transmission rate.

To ensure fairness in user communication and improve system resource utilization, this paper proposes a clustering strategy based on distributed RIS-assisted CoMP user grouping. The AGD clustering algorithm is employed to match CoMP users with RISs for grouping, thereby achieving more efficient signal enhancement with distributed RISs.

During inter-group power optimization allocation, the coordination between NOMA user clusters and inter-group power allocation is established. A user-grouping scheme based on channel strength is proposed to ensure minimal inter-group channel correlation. Ultimately, closed-form expressions for inter-group power allocation and coordinated NOMA clustering are derived to obtain the optimal solution.

The rest of this article is organized as follows: Section 2 introduces the system model and transmission signals of the article. Section 3 discusses the deployment of RISs and user grouping based on the distributed RISs. Section 4 allocates power between groups and within groups and describes the complexity analysis. Section 5 presents the simulation results and analysis. Section 6 presents the conclusion and future prospects of the article.

## 2. System Model

**Figure 1:**
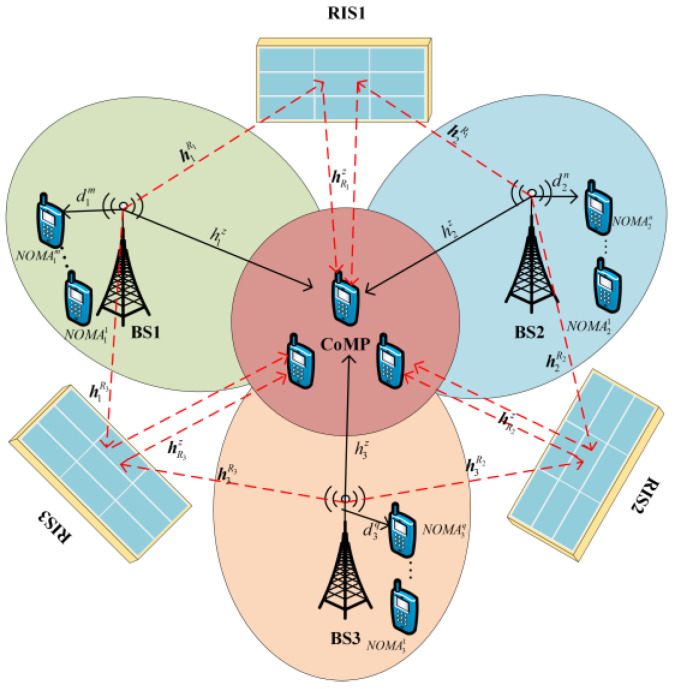
System model.

Consider the distributed RIS-assisted multi-cell CoMP-NOMA transmission network depicted in Figure 1, where the system comprises three base stations (expandable to multiple base stations), K users, and three intelligent reflecting surfaces. In this framework, two main user categories are considered: NOMA users and CoMP users. NOMA users communicate with a single base station, while CoMP users receive signal transmissions from all three base stations under CoMP transmission scheduling. Distributed RISs are deployed around the clustered area of CoMP users, with each RIS being composed of L reflecting units. By adjusting the phase and amplitude of the signal, the RIS reflects electromagnetic wave signals in predetermined directions and magnitudes, thereby enhancing the signals for CoMP users.

In the system model shown in Figure 1, it is assumed that there is a transmitting antenna and a receiving antenna between the base station and cellular users; set the number of NOMA users for services BS_1_, BS_2_, and BS_3_ to M, N, and Q, respectively, and the user set is represented as m∈[1,2,⋯,M], n∈[1,2,⋯,N], q∈[1,2,⋯,Q]. The number of CoMP users jointly served by all three base stations is denoted as Z, and the set of CoMP users is denoted as z∈[1,2,⋯,Z]. In the joint user grouping described in this paper, a cluster is defined as a cellular user cluster composed of three NOMA users and one CoMP user, represented as $C_{i}=\left[NOMA_{1}^{m},NOMA_{2}^{n},NOMA_{3}^{q},CoMP^{z}\right]$.

To address the issue of uneven distribution in the allocation of NOMA users and CoMP users, a CoMP user can exist in one or more clusters, and each cluster must have at least one NOMA user.

### 2.1. Signal Model

To enhance channel gains, RISs are deployed above the area where CoMP users are concentrated. Assuming three base stations serving their respective NOMA users, the distances between each base station and the NOMA users they serve are $d_{1}^{m}$, $d_{2}^{n}$, and $d_{3}^{q}$. To the CoMP users, the direct distances are $h_{1}^{z}$, $h_{2}^{z}$, and $h_{3}^{z}$. The channels to the RISs are $h_{1,R}$, $h_{2,R}$, and $h_{3,R}$. The reflected channel from the RIS to the CoMP users is $h_{R,Co}$. The communication between the base station and the users is influenced by various factors such as reflection, diffraction, etc., making it prone to Rayleigh fading. Therefore, the channels from the base station to the CoMP users and NOMA users are represented using the Kronecker model:

(1) $$h_{Co}^{B\to Co}=\frac{\sqrt{\psi_{B\to R}}\xi_{Co}^{B\to Co}}{{\left(d_{Co}^{B\to Co}\right)}^{\frac{\eta_{B\to Co}}{2}}},$$

(2) $$h_{NO}^{B\to NO}=\frac{\sqrt{\psi_{B\to R}}\xi_{NO}^{B\to NO}}{{\left(d_{NO}^{B\to NO}\right)}^{\frac{\eta_{B\to NO}}{2}}},$$

where $d_{Co}^{B\to Co}$, $d_{NO}^{B\to NO}$ represent the distances between the base station and CoMP users, and between the base station and NOMA users, respectively; $\eta_{B\to Co}$, $\eta_{B\to NO}$ are the path loss exponents for the direct path between the base station and users; and $\psi_{B\to R}$ represents the covariance matrix received by the base station. Each element in $\xi_{Co}^{B\to Co}$ and $\xi_{NO}^{B\to NO}$ follows a complex Gaussian distribution with zero mean and unit variance.

The primary path from the base station to the RIS is the line-of-sight (LOS) path, exhibiting mainly Rician fading. Therefore, the channel from the base station to the RIS can be represented as follows:

(3) $$G_{B\to R}=\frac{{\left(\frac{\kappa_{B\to R}}{\kappa_{B\to R}+1}\right)}^{\frac{1}{2}}{\bar{\xi }}_{B\to R}}{{\left(d_{B\to R}\right)}^{\frac{\eta_{B\to R}}{2}}}+\frac{{\left(\frac{1}{\kappa_{B\to R}+1}\right)}^{\frac{1}{2}}\sqrt{\psi_{B\to R}}\xi_{B\to R}\sqrt{\psi_{R}}}{{\left(d_{B\to R}\right)}^{\frac{\eta_{B\to R}}{2}}},$$

where $d_{B\to R}$ represents the distance between the base station and the RIS, $\kappa_{B\to S}$ represents the Rician-K factor between the base station and the RIS, and $\eta_{B\to R}$ represents the loss exponent for the distance between the base station and the RIS. ${\bar{\xi }}_{B\to R}$ is the LOS component between the base station and the RIS, where each element in $\xi_{B\to R}$ follows a complex Gaussian distribution with zero mean and unit variance. $\psi_{R}$ represents the reflection correlation matrix at the RIS. Similarly, the channel model between the RIS and CoMP users follows a Rician fading model and can be expressed as follows:

(4) $$g_{R\to Co}=\frac{{\left(\frac{\kappa_{R\to Co}}{\kappa_{R\to Co}+1}\right)}^{\frac{1}{2}}{\bar{\zeta }}_{R\to Co}}{{\left(d_{R\to Co}\right)}^{\frac{\eta_{R\to Co}}{2}}}+\frac{{\left(\frac{1}{\kappa_{R\to Co}+1}\right)}^{\frac{1}{2}}\sqrt{\psi_{R\to Co}}\zeta_{R\to Co}}{{\left(d_{R\to Co}\right)}^{\frac{\eta_{R\to Co}}{2}}}.$$

where $d_{R\to Co}$ represents the distance between the RIS and the CoMP user, $\eta_{R\to Co}$ represents the loss exponent between the RIS and the CoMP user, ${\bar{\zeta }}_{R\to Co}$ represents the LOS component, and $\psi_{R\to Co}$ represents the receive correlation matrix at the RIS.

### 2.2. Analysis of Rates

The transmission of signals from the three base stations to the NOMA users and CoMP users according to the NOMA principle can be represented as follows:

(5) $$y_{NO,Co}^{t}=\sqrt{\alpha_{c,NO}^{i}P_{t}}y_{NO}^{t}+\sqrt{\alpha_{c,Co}P_{t}}y_{Co}^{t},$$

where t∈[1,2,3]; $P_{t}$ represents the transmission power of the t-th base station; $\alpha_{c,NO}^{i}$ represents the power allocation factor for the i-th NOMA user in the c-th group; $\alpha_{c,Co}$ represents the power allocation factor for the c-th group of CoMP users, and satisfies $0≺\alpha_{c,NO}^{i}≺\alpha_{c,Co}≺1$; and $y_{NO}^{t}$ and $y_{Co}^{t}$, respectively, represent the desired signals received by NOMA users and CoMP users at the n-th base station.

Due to severe obstacles blocking the path and path attenuation caused by long-distance transmission, we neglect signals with two or more reflections as well as interference from neighboring cells on NOMA users. The signals received by NOMA users served by each base station are as follows:

(6) $${\tilde{y}}_{NO,m}^{1}=d_{1}^{m}y_{NO,Co}^{1}+\omega_{NO,m}^{1},$$

(7) $${\tilde{y}}_{NO,n}^{2}=d_{2}^{n}y_{NO,Co}^{2}+\omega_{NO,n}^{2},$$

(8) $${\tilde{y}}_{NO,q}^{3}=d_{3}^{q}y_{NO,Co}^{3}+\omega_{NO,q}^{3},$$

where $\omega_{NO,m}^{1}$, $\omega_{NO,n}^{2}$, and $\omega_{NO,q}^{3}$ represent the Gaussian white noise received by the NOMA users.

In the transmission of the CoMP-NOMA network framework, CoMP users receive signals from three base stations, and the signal expression is as follows:

(9) $$\begin{array}{l} {\tilde{y}}_{Co,z}=\left(h_{1}^{z}+h_{1,R}\Phi_{s}h_{R,Co}\right)y_{NO,Co}^{1}+\\ \;\;\;\;\left(h_{2}^{z}+h_{2,R}\Phi_{s}h_{R,Co}\right)y_{NO,Co}^{2}+\\ \;\;\;\;\left(h_{3}^{z}+h_{3,R}\Phi_{s}h_{R,Co}\right)y_{NO,Co}^{3}+\omega_{Co,z} \end{array}$$

where $\omega_{Co,z}$ represents the Gaussian white noise received by the CoMP user, and $\Phi_{s},s\in \left\{1,2,3\right\}$ is the phase-shift matrix, defined below:

(10) $$\Phi_{s}=diag\left\{\Phi_{s}^{1},\Phi_{s}^{2},\cdots ,\Phi_{s}^{L}\right\},$$

where $\left\{\Phi_{s}^{L}=e^{j\theta_{l}},0\leq \theta_{l}\leq 2\pi \right\}$.

Low-power transmission is a crucial step in the close integration of green development and mobile communication. To achieve fairness in communication transmission, NOMA users in cells with better channel conditions typically consume less transmission power than CoMP users. In the user demodulation order of this paper, NOMA users have a priority SIC decoding order. When demodulating NOMA user signals, CoMP user signals are treated as noise. Therefore, the Signal-to-Interference-plus-Noise Ratio (SINR) for the NOMA users in the cluster ($\left[c,NO_{i}\right],i\in \left\{1,2,3\right\}$) can be expressed as follows:

(11) $$\gamma_{c,NO_{i}}=\frac{\varepsilon_{c}\alpha_{c,NO}^{i}P{\left|h_{t}^{i}\right|}^{2}}{I_{c}+I_{c’}+\sigma^{2}}.$$

In the above equation, $\varepsilon_{c}$ is the inter-group power allocation factor for the c-th group, where $d_{t}^{i}$ represents the distance from the t-th base station to the i-th NOMA user; $\sigma^{2}$ is the Gaussian white noise power; and P is the total transmission power, which can be expressed as $P=P_{1}+P_{2}+P_{3}$. $I_{c}$ and $I_{c’}$ are the intra-group interference and inter-group interference, respectively, which can be expressed as $I_{c}=\sum_{i’=1,i’\neq i}^{3}\varepsilon_{c}\alpha_{c}^{i}P{\left|d_{t}^{i’}\right|}^{2}+\varepsilon_{c}\left(1-\alpha_{c}^{i}\right)P{\left|H_{c,Co}\right|}^{2}$, $I_{c’}=\sum_{c’=1,c’\neq c}^{C}\varepsilon_{c’}P{\left|H_{c’,Co}+D_{c’,Co}\right|}^{2}$, where $\varepsilon_{c’}$ is the inter-group power allocation factor for the C’-th group, $H_{C,Co}=h_{t}^{z}+h_{t,R}\Theta_{s}h_{R,Co}$, $D_{C,Co}=d_{1}^{m}+d_{2}^{n}+d_{3}^{q}$.

After the successful decoding of all NOMA users, CoMP users will remove the signals of NOMA users and then decode their signals. The SINR for the C-th group of CoMP users ($\left[c,Co\right]$) is given in the following equation:

(12) $$\gamma_{c,Co}=\frac{\varepsilon_{c}\alpha_{c,Co}P{\left|H_{c,Co}\right|}^{2}}{I_{c’}+\sigma^{2}}.$$

## 3. User Grouping Based on Distributed RISs

In the deployment of multiple RISs in this article, the deployment of RIS1 should consider the relative positional relationship between BS_1_, BS_2_, and CoMP users. Calculate the centroid of the CoMP user cluster ${ο}_{C}=\frac{\sum_{n}^{\left|C_{n}\right|}Z_{C,n}}{\left|C_{n}\right|}$, where the distance between BS_1_ and ${ο}_{C}$ is $D_{1}$, the distance between BS2 and ${ο}_{C}$ is $D_{2}$, and the vertical distances from RIS1 to BS_1_-${ο}_{C}$ and BS2-${ο}_{C}$ are $T_{1}$ and $T_{2}$, respectively. Theorem 1 in reference [30] states the following: 

When $T_{1}≺\frac{D_{1}}{2}$ or $T_{2}≺\frac{D_{2}}{2}$, the RIS should be deployed near UE to maximize the received signal power.

When $T_{1}\geq \frac{D_{1}}{2}$ or $T_{2}\geq \frac{D_{2}}{2}$, RIS1 should be deployed at the intersection of the perpendicular lines in BS_1_-Oc and BS_2_-Oc to maximize the received signal power.

Similarly, we can complete the deployment of RIS2 and RIS3.

From a fairness perspective, our goal is to cluster users with similar channel conditions using clustering strategies to obtain optimal RIS-enhanced signals and improve service fairness. In this paper, we employ the AGD clustering algorithm for edge user clustering. According to the system model, CoMP users are clustered into three clusters ($g_{1}$, $g_{2}$, $g_{3}$), with each cluster assisted with an intelligent reflecting surface for communication.

First, we address the distribution of edge users within the plane. By computing the centroid of the network based on user coordinates, once the centroid coordinates are determined, we connect the centroid point with the coordinates of adjacent base stations, thereby delineating the angular range for each clustered user. Subsequently, scanning begins based on the centroid. When a user falls within the angular range formed by the centroid and points BS_1_ and BS_2_, they are assigned to the cluster $g_{1}$, and the scanning process continues for the next user. Additionally, we do not consider dynamic errors caused by user mobility, and each user can only be assigned to one user group.

We propose a strategy to match clustered users with the most suitable RIS by utilizing the minimum average distance between the centroid of the search user cluster and the centroid of a single RIS, thereby achieving the purpose of assisted communication. According to the known coordinates of the $g_{j},j\in \left\{1,2,3\right\}$ user within the n-th cluster $Z_{C,n}={\left[x_{C,n},y_{C,n}\right]}^{T},\forall n\in \left[1,\cdots |C_{n}|\right]$, the centroid of the user cluster is given as ${ο}_{C}=\frac{\sum_{n}^{\left|C_{n}\right|}Z_{C,n}}{\left|C_{n}\right|}$, using binary variables $A_{c,l}\in \left\{0,1\right\}$. To indicate that the j-th cluster selects the l-th RIS for communication, we define an RIS selection matrix as follows:

(13) $$A=\left[\begin{array}{ccc} A_{1,1} & \cdots & A_{1,L}\\ \vdots & \ddots & \vdots \\ A_{C,1} & \cdots & A_{C,L} \end{array}\right]$$

The objective function for obtaining the minimum sum distance using a binomial norm is represented as follows:

(14) $$\begin{array}{l} \underset{A}{\min}\sum_{c=1}^{C}\sum_{l=1}^{L}A_{c,l}||\lambda_{c}-\mu_{l}\left|\right|\\ s.t.\left(1\right)\;rank(A)=C\\ \;\;\left(2\right)A_{c,l}\in \left\{0,1\right\},\forall c\in \left[1,\cdots C\right],\forall l\in \left[1,\cdots L\right]\;\\ \;\;\left(3\right)\sum_{l=1}^{L}A_{c,l}=1,\forall c\in \left[1,\cdots C\right],\forall l\in \left[1,\cdots L\right] \end{array}$$

where $\left(1\right)$ is a full rank constraint, $\left(2\right)$ ensures that each RIS block is in a working state, and $\left(3\right)$ ensures that each cluster is only served by one RIS.

## 4. Power Allocation Scheme

By incorporating green communication principles and implementing low-power technologies, we aim to promote sustainable development and reduce the amount of energy resources consumed by communication systems. While ensuring that each user’s minimum QoS requirements are met, the optimization problem can be formulated as presented below:

(15) $$\begin{array}{l} \underset{\varepsilon_{c},\Phi_{s},\alpha_{c,NO}^{i},\alpha_{c,Co},UC}{\min}P=\sum_{c=1}^{C}\left({\sum_{i=1}^{I}\left(\alpha_{c,NO}^{i}\right)}^{2}+{\left(\alpha_{c,Co}\right)}^{2}\right)\\ s.t\;C1:\gamma_{c,NO_{i}}\geq \min\gamma_{c,NO_{i}}\\ \;\;\;C2:\gamma_{c,Co}\geq \min\gamma_{c,Co}\\ \;\;\;C3:|\Phi_{l}|=1,1\leq l\leq L \end{array}$$

where $\varepsilon_{c}$, $\Phi_{s}$, $\alpha_{c,NO}^{i}$, $\alpha_{c,Co}$, and UC are the optimization variables, and C1 and C2 ensure the minimum QoS for NOMA users and CoMP users, representing the single-mode constraint for each phase shifter.

The objective function involves multiple optimization variables, making it difficult to solve directly. To address this challenge, this paper approaches the problem from two perspectives: inter-group power allocation and intra-group power allocation, aiming to minimize the total transmission power. The optimization of inter-group power allocation is achieved through the joint grouping of CoMP-NOMA users, while the optimization of intra-group power allocation is accomplished through the phase-shift design of the RISs.

### 4.1. Joint Inter-Group PA and Coordinated NOMA UC Optimization

This section is dedicated to finding an optimal joint CoMP-NOMA user-grouping strategy. Through inter-group power allocation, the objective is to maximize the sum-rate at the group level.

When employing NOMA transmission and SIC detection, a higher sum-rate can be achieved when there is significant channel diversity between groups. Considering this, when forming joint CoMP-NOMA user groups, the inter-group user channel correlation should be sufficiently low. As shown in Algorithm 1, we perform joint NOMA user clustering.
**Algorithm 1** Coordinated NOMA user cluster algorithm.**Input** the channel status of NOMA users and CoMP users served by each of the three base stations $g_{1}^{x}=\left\{h_{1}^{1},h_{1}^{2}\cdots h_{1}^{x}\right\},$   $g_{2}^{y}=\left\{h_{2}^{1},h_{2}^{2}\cdots h_{2}^{y}\right\},$ $g_{3}^{z}=\left\{h_{3}^{1},h_{3}^{2}\cdots h_{3}^{z}\right\},$ $g_{4}^{r}=\left\{H_{Co}^{1},H_{Co}^{2}\cdots H_{Co}^{r}\right\}$$,\;C_{i};$ **initializing** $C_{i}[0]=0;$
 **repeat** 

  Dynamic programming iteration: Outer loop iterates through
$g_{e},e\in \left\{1,2,3,4\right\}$, inner loop iterates through each element within the current group;

   **if** the current state can reach;

    Update the next status value $C_{i+1};$
  **repeat**

   Calculate the sum of all combinations
$C_{total};$
   **for** from 0 to
$\frac{C_{total}}{2};$
    Find the value closest to half of the total as the target value for the optimal solution
$C_{optimal};$
 **repeat**

   Traverse the elements in each group and determine whether to select the cur-rent element based on the values in the dynamic programming array;

   **if** selected the current element, then add it to the
$C_{optimal}$, update $C{’}_{total};$
  **until** all users participate in completing the grouping, with at least one NOMA user and CoMP user in each group;

 **Output** grouping results
$C_{i},i\in \left\{1,2,\cdots ,C\right\}$.

Due to the introduction of RISs, the channels of CoMP users are synthesized from direct channels and reflected channels. In theory, when optimizing inter-group power and joint CoMP-NOMA user grouping, the phase shifts introduced by the RISs should be considered. However, to reduce complexity, the phase-shift factor of the RISs is not considered during the user-grouping process. The inter-group channel correlation is defined as follows:

(16) $$H_{c,c’}=\frac{\left|{H_{c}}^{H}H_{c’}\right|}{\left\|H_{c}\right\|\left\|H_{c’}\right\|}.$$

where $H_{c}=h_{1}^{c}+h_{2}^{c}+h_{3}^{c}+H_{c,Co}$ represents the channel state of the c-th group. Considering the users in the c-th group as a whole, the SNR can be expressed as follows:

(17) $$\gamma_{c}=\frac{{\left|H_{c}\right|}^{2}\varepsilon_{c}P}{\sum_{c’=1,c’\neq c}^{C}{\left|H_{c’}\right|}^{2}\varepsilon_{c’}P+\sigma^{2}}.$$

The achievable rate of the C-th group is

(18) $$R_{c}=\log_{2}\left(1+\gamma_{c}\right).$$

On a group basis, the reachable sum-rate of the system is

(19) $$R_{sum}=\sum_{c=1}^{C}\log_{2}\left(1+\frac{{\left|H_{c}\right|}^{2}\varepsilon_{c}P}{\sum_{c’=1,c’\neq c}^{C}{\left|H_{c’}\right|}^{2}\varepsilon_{c’}P+\sigma^{2}}\right).$$

The greater the channel gain differences between users, the more significant the throughput improvement of the NOMA system will be compared to the OMA system. The optimization problem for inter-group power allocation can be formulated as follows:

(20) $$\begin{array}{l} \max\;R_{sum}=\sum_{c=1}^{C}R_{c}\\ s.t\;C4:R_{c}\geq R_{\min},\\ \;\;C5:P\geq 0\\ \;\;C6:P\leq P_{\max} \end{array}$$

where C4 ensures that the users in the c-th group satisfy the minimum QoS. Directly solving the optimization problem in Equation (17) requires traversing all possible user-grouping scenarios, resulting in extremely high complexity. Next, a simplified approach to this optimization problem is presented.

From Equation (16), the following can be obtained:

(21) $$\varepsilon_{c}=\frac{\gamma_{c}\left(\sum_{c’=1,c’\neq c}^{C}{\left|H_{c’}\right|}^{2}\varepsilon_{c’}P+\sigma^{2}\right)}{{\left|H_{c}\right|}^{2}P}.$$

It can be observed that the higher the channel gain of the c-th group, the smaller its power allocation factor $\varepsilon_{c}$. Furthermore, Equation (22) can be derived:

(22) $$\varepsilon_{c}=\gamma_{c}\frac{\left(1-\varepsilon_{c}\right){\left|H_{r}\right|}^{2}}{{\left|H_{c}\right|}^{2}}+\frac{\gamma_{c}\sigma^{2}}{{\left|H_{c}\right|}^{2}P},$$

where ${\left|H_{r}\right|}^{2}=\sum_{c’=1,c’\neq c}^{C}{\left|H_{c’}\right|}^{2}$. Through transformation, we obtain

(23) $$\varepsilon_{c}=\frac{P\gamma_{c}{\left|H_{r}\right|}^{2}+\gamma_{c}\sigma^{2}}{P\left({\left|H_{c}\right|}^{2}+\gamma_{c}{\left|H_{r}\right|}^{2}\right)}.$$

Using Equation (17), the correlation coefficient between the channel gain of the c-th group and the interference from other groups’ channel gains can be defined as follows:

(24) $$H_{c,r}=\frac{\left|{H_{c}}^{H}H_{r}\right|}{\left\|H_{c}\right\|\left\|H_{r}\right\|}$$

From Equation (22) to Equation (23), it can be inferred that when the channel gain of a particular group is determined, the power allocation factor for that group can be obtained. After the joint CoMP-NOMA user grouping is completed, and the inter-group channel states are known, the power allocation between channels can be achieved.

### 4.2. Alternating Optimization of Intra-Group PA and PS Design

After the inter-group power allocation is completed, fixed power levels are obtained for each group of users $p_{c}=\varepsilon_{c}P$. According to Equations (12) and (13), the achievable rates for NOMA users and CoMP users within the group can be determined as follows:

(25) $$R_{c,NO}=\sum_{i=1}^{I}\log_{2}\left(1+\gamma_{c,NO_{i}}\right),$$

(26) $$R_{c,Co}=\log_{2}\left(1+\gamma_{c,Co}\right).$$

The achievable rates for the c-th group are

(27) $$R_{c}^{NO,Co}=R_{c,NO}+R_{c,Co}.$$

The intra-group power allocation in this paper aims to maximize the achievable rates for users within the group. The optimization problem is formulated as presented below:

(28) $$\begin{array}{l} \max R_{c}^{NO,Co}\\ s.t\;C7:0≺\alpha_{c,NO}^{i}≺\alpha_{c,Co}≺1\\ \;\;C8:0\leq p\left(\alpha_{c,NO}^{i}+\alpha_{c,Co}\right)\leq p_{c}\\ \;\;C9:R_{c,NO_{i}}\geq R_{c,NO_{i}}^{\min}\\ \;\;C10:R_{c,Co}\geq R_{c,Co}^{\min}\\ \;\;C11:\left|\Phi_{l}\right|=1,1\leq l\leq L, \end{array}$$

where C7 ensures that CoMP users within the group can achieve better service quality; C8 guarantees that the sum of allocated power within the group does not exceed the fixed power for each group $p_{c}$; and C9 and C10 ensure that users within the group receive at least the minimum QoS. In this optimization objective, the intra-group power allocation is highly coupled with the phase shift, making it challenging to directly obtain the optimal solution. In the following, this paper will adopt an alternating optimization approach to solve the coupling problem between the power and phase shift.

Initializing the phase shift $\Phi_{s}^{\left(0\right)}$, in the case of a fixed phase shift, the channel of CoMP users is known as $H_{C,Co}=h_{t}^{z}+h_{t,R}\Theta_{s}h_{R,Co}$, and the objective function for power allocation optimization can be expressed using intra-group power allocation coefficients:

(29) $$\begin{array}{l} \min\sum_{i=1}^{I}{\left(\alpha_{c,NO}^{i}\right)}^{2}+{\left(\alpha_{c,Co}\right)}^{2}.\\ s.t\;C7,C9,C10 \end{array}$$

This objective function is convex and can be solved using CVX. When the optimal power allocation coefficients ${\hat{\alpha }}_{c,NO}^{i}$ and ${\hat{\alpha }}_{c,Co}$ are obtained in the t-th iteration, the equality holds in

(30) $${\left({\hat{\alpha }}_{c,NO}^{i}\right)}^{2}=\frac{\left(2^{R_{c,NO_{i}}^{\min}}-1\right)\left(I_{c}+I_{c’}+\sigma^{2}\right)}{{\left|h_{t}^{i}\right|}^{2}},$$

(31) $${\left({\hat{\alpha }}_{c,Co}\right)}^{2}=\frac{\left(2^{R_{c,Co}^{\min}}-1\right)\sigma^{2}}{{\left|H_{c,Co}\right|}^{2}}.$$

When obtaining the fixed power allocation factors for intra-group users during the initialization of the phase shift, optimizing the phase shift to maximize the achievable rates for CoMP users can be formulated as the optimization objective function:

(32) $$\begin{array}{l} \max\;R_{c,Co}^{sum}=\sum_{c=1}^{C}\log_{2}\left(1+\frac{p{\hat{\alpha }}_{c,Co}{\left|H_{c,Co}\right|}^{2}}{\sigma^{2}+I_{c’}}\right),\\ s.t\;C7,C9,C10,C11. \end{array}$$

If we let $w_{c}={\left|H_{c,Co}\right|}^{2}={\left|h_{t}^{z}+h_{t,R}\Theta_{s}h_{R,Co}\right|}^{2}$, C10 can be rewritten as follows:

(33) $$w_{c}\geq \frac{\partial_{c}\left(2^{R_{c,Co}^{\min}}-1\right)}{{\hat{\alpha }}_{c,Co}}$$

where $\partial_{c}=\frac{\sigma^{2}+I_{c’}}{p}$. Introducing auxiliary variables provides the following:

(34) $$\Theta_{s}^{c}=\left[\begin{array}{cc} x_{c}{x_{c}}^{H} & x_{c}h_{t}^{z}\\ {h_{t}^{z}}^{H}{x_{c}}^{H} & 0 \end{array}\right]$$

where $x_{c}=diag\left({h_{t,R}}^{H}\right)h_{R,Co}$, and ${\left|h_{t}^{z}+h_{t,R}\Theta_{s}h_{R,Co}\right|}^{2}$ can be rewritten as ${\left|h_{t}^{z}+{\Phi_{l}}^{H}x_{c}\right|}^{2}$. Then, $\gamma_{c,Co}$ can be rewritten as

(35) $$\gamma_{c,Co}=\frac{\nu_{x_{c}}}{\mu_{x_{c}}},$$

where $\nu_{x_{c}}$ and $\mu_{x_{c}}$ can be expressed as follows:

(36) $$\nu_{x_{c}}={\left|h_{t}^{z}+{\Phi_{l}}^{H}x_{c}\right|}^{2}{\hat{\alpha }}_{c,Co}p$$

(37) $$\mu_{x_{c}}=\sum_{c’=1,c’\neq c}^{C}\varepsilon_{c’}P{\left|h_{t}^{z}+{\Phi_{l}}^{H}x_{c}+D_{c’,Co}\right|}^{2}+\sigma^{2}$$

According to the quadratic transformation, the objective function in Equation (30) can be transformed into the following:

(38) fxc,γ=∑c=1C2α^c,CoReγc∗htz+ΦlHxc−∑c=1Cγc2∑c’=1,c’≠cCεc’Phtz+ΦlHxc+Dc’,Co2+σ2

where $\gamma =\left[\gamma_{1},\gamma_{2},\cdots ,\gamma_{C}\right]$ is a Lagrangian auxiliary variable, Re is the real part taken, and ∗ is the taking conjugation.

The optimization problem in Equation (32) can be rewritten as

(39) $$\begin{array}{l} \underset{x_{c},\gamma }{\max}\sum_{c=1}^{C}f\left(x_{c},\gamma \right)\\ s.t.\;C12:w_{c}\geq \frac{\partial_{c}\left(2^{R_{c,Co}^{\min}}-1\right)}{{\hat{\alpha }}_{c,Co}},\\ \;\;\;C13:\left|\Phi_{l}\right|=1,1\leq l\leq L \end{array}$$

If we let $\frac{\partial f\left(x_{c},\gamma \right)}{\partial \gamma_{c}}=0$, and $\frac{\partial f\left(x_{c},\gamma \right)}{\partial x_{c}}=0$, the optimal solution for γ can be obtained as follows:

(40) $$\tilde{\gamma }=\frac{\sqrt{{\hat{\alpha }}_{c,Co}}\left(h_{t}^{z}+{\Phi_{l}}^{H}x_{c}\right)}{\sum_{c’=1,c’\neq c}^{C}\varepsilon_{c’}P{\left|h_{t}^{z}+{\Phi_{l}}^{H}x_{c}+D_{c’,Co}\right|}^{2}+\sigma^{2}}.$$

The optimal solution for $x_{c}$ obtained using the Lagrange multiplier method is

(41) $${\tilde{x}}_{c}=\frac{\sqrt{{\hat{\alpha }}_{c,Co}}\gamma_{c}H_{j}}{\sum \chi E_{L}+\sum_{j=c}^{C}{\left|\gamma_{j}\right|}^{2}H_{j}{H_{j}}^{H}},$$

where $E_{L}$ is the L-order identity matrix, and χ is the auxiliary variable that satisfies the constraint conditions. In summary, the alternating optimization method for power allocation and phase shift within the group is shown in Algorithm 2:
**Algorithm 2** Alternating optimization of intra-group PA and PS. **Input**$\;p^{\left(0\right)}$$,\;{\Phi^{1}}^{\left(0\right)}$$,{\Phi^{2}}^{\left(0\right)}$$,{\Phi^{3}}^{\left(0\right)}$$,\;{\alpha_{c,Co}}^{\left(0\right)}$$,\;{\alpha_{c,NO}^{i}}^{\left(0\right)}$, the maximum number of iterations is *T* and the system’s initial sum-rate is $R_{sum}^{\left(t\right)}=0$. Set the current iteration number to *t* = 0; **While** (t≤T$\;\mathrm{or}\;R_{sum}^{\left(t+1\right)}-R_{sum}^{\left(t\right)}≻\varsigma )$  **repeat**

   Fix
${\Phi^{1}}^{\left(0\right)}$$,\;\mathrm{update}\;{\alpha_{c,Co}}^{\left(1\right)}$$\;\mathrm{and}\;{\alpha_{c,NO}^{i}}^{\left(1\right)}$ through Equation (29);

   Fix
${\alpha_{c,Co}}^{\left(1\right)}$$,\;{\alpha_{c,NO}^{i}}^{\left(1\right)}$$,\;\mathrm{update}\;\Phi_{s}^{\left(1\right)}$ through Equation (39);

  **until** (
t=T or $R_{sum}^{\left(t+1\right)}-R_{sum}^{\left(t\right)}\leq \varsigma$) ${\hat{\Phi }}_{s}^{\left(t\right)}={\hat{\Phi }}_{s}^{\left(t+1\right)}$, ${{\hat{\alpha }}_{c,Co}}^{\left(t\right)}={{\hat{\alpha }}_{c,Co}}^{\left(t+1\right)}$, ${{\hat{\alpha }}_{c,NO}^{i}}^{\left(t\right)}={{\hat{\alpha }}_{c,NO}^{i}}^{\left(t+1\right)};$ **Output** ${\hat{\Phi }}_{s}^{\left(t\right)}$, ${{\hat{\alpha }}_{c,Co}}^{\left(t\right)}$, ${{\hat{\alpha }}_{c,NO}^{i}}^{\left(t\right)}$.

### 4.3. Complexity Analysis

The complexity analysis presented in this article mainly includes the following two aspects: user grouping based on distributed RISs, and grouping COMP users and matching them with the best-matching RISs for auxiliary communication, with a complexity of $O\left(Z^{2}T\right)$. To quantify the computational complexity of the distributed RIS-assisted COMP-NOMA system based on cluster strategy and the AO algorithm designed for rate maximization proposed in Algorithm 2, it is necessary to evaluate the computational complexity of PA-UC optimization and PA-PS optimization. The computational complexity of the optimal reachable sum of all possible coordinated NOMA cluster configurations for PA-UC is $O\left(MNQZ\right)$. PA-PS optimization requires multiple replacements of the Lagrange multipliers, and its complexity is $O\left(L^{3}\right)$. Therefore, the overall complexity of the AO algorithm is $O\left(J_{1}MNQZ+J_{2}L^{3}\right)$. In summary, the time complexity of the algorithm proposed in this article is $O\left(Z^{2}T+J_{1}MNQZ+J_{2}L^{3}\right)$.

## 5. Simulation Results

Through the analysis of simulation experiment data, this section validates the communication performance of the proposed energy-efficient RIS-assisted coordinated multi-point transmission NOMA system user-grouping design. In a three-dimensional plane, we assume that three small-cell base stations are located at BS_1_ (1000,2000), BS_2_ (2500,2000), and BS_3_ (1750,1000). Three RISs are located separately at RIS1 (1750,3000), RIS2 (1000,1000), and RIS3 (2500,1000). To meet the basic QoS for users, we set $\gamma_{\min}=-10\;\mathrm{dB}$. More parameters are given in Table 2.

**Figure 2:**
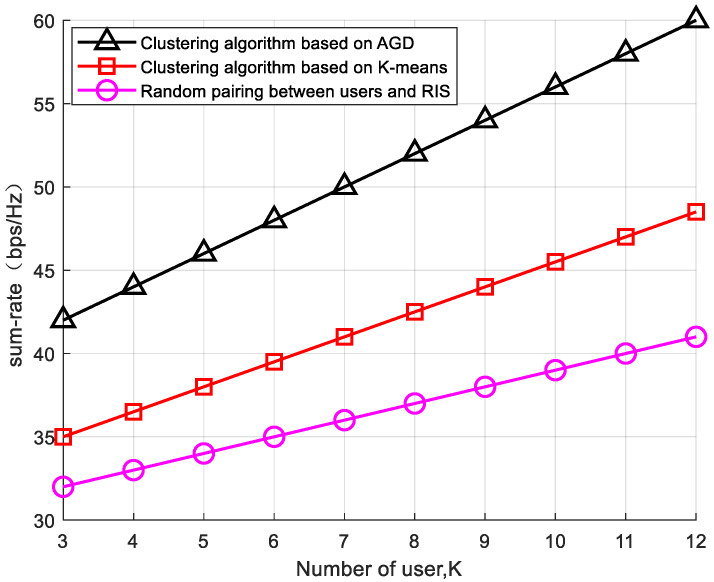
The impact of RIS user grouping on the system, L = 25.

To validate the enhancement of overall system performance using the RIS-based edge user-grouping strategy, experiments were conducted to compare the impacts of different clustering algorithms and user–RIS pairings on the system’s performance and rates. With a fixed number of reflecting elements (L = 25) and a system transmission power of P = 30 dB, as shown in Figure 2, the simulation results indicate the impact of user numbers on the data transmission rate of mobile communication networks. As the number of users increases, the system faces more communication demands, leading to an increase in the data transmission rates. The AGD clustering algorithm adopted in this paper demonstrates the best performance enhancement in terms of system and rate. The K-means clustering algorithm clusters users with similar Euclidean distances into the same group, and the distribution of users and RISs greatly affects the clustering effect. In situations where user distribution is uneven, this can lead to small channel differences within each cluster and an uneven distribution of users. When users randomly select an RIS for assistance communication, the system performance is severely degraded due to geographical location and channel fading effects. Therefore, grouping edge users and making reasonable matches with RISs can effectively enhance the system’s overall rate.

**Figure 3:**
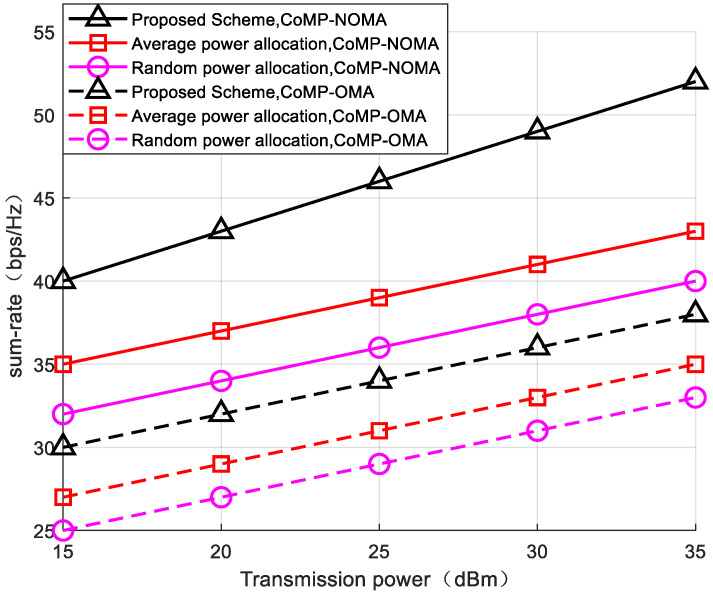
The relationship between the system’s rate and transmission power, L = 25.

To verify the improvement in the system sum-rate achieved by optimizing the inter-group power allocation and employing a joint CoMP-NOMA user-grouping strategy, experiments were conducted with a fixed number of reflecting elements (L = 25) comparing the following scenarios: (1) equal inter-group power allocation, and (2) random inter-group power allocation. As depicted in Figure 3, as the power level increases, the signal coverage range expands, and users can receive signals in a wider area. Moderately increasing the power can improve the transmission quality, reduce signal loss and interference, and thus improve the communication efficiency and data transmission rate. Under different power gains, the algorithm proposed in this paper demonstrates superior enhancement in the system’s sum-rate within the CoMP-NOMA system compared to other approaches. When inter-group power is equally allocated, due to significant channel variations among inter-group users, ensuring the minimum service quality for some user groups may be challenging. Consequently, equal power allocation among user groups in the presence of channel disparities violates communication fairness, thus affecting the overall system throughput. OMA schemes, unaffected by bandwidth reuse gains, require more transmit power to achieve the same sum-rate.

**Figure 4:**
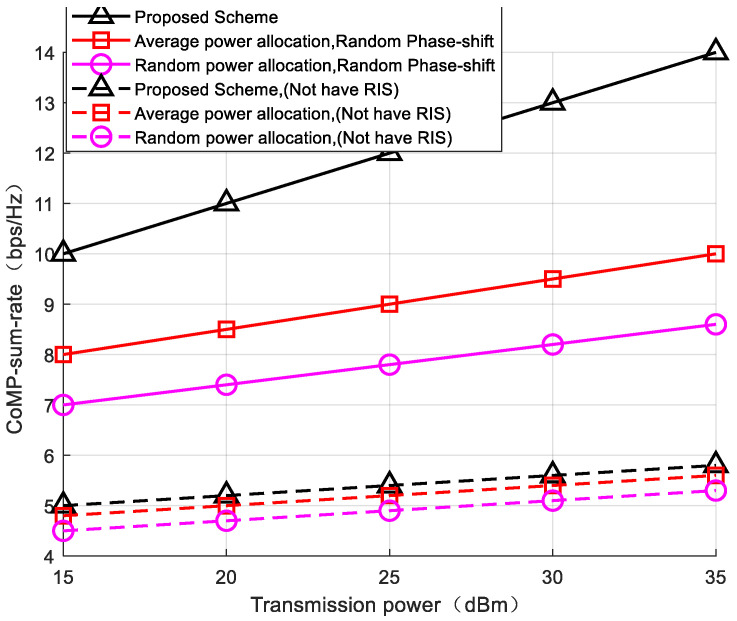
The relationship between CoMP users, rate, and transmission power.

To validate the enhancement in the system sum-rate for CoMP users achieved through the alternating optimization of intra-group power allocation and phase shift, experiments were conducted with a fixed number of reflecting elements (L = 25), comparing the following scenarios: (1) equal intra-group power allocation with random phase-shift control, and (2) random intra-group power allocation with random phase-shift control. As shown in Figure 4, with the increase in system power gain, the sum-rate of CoMP users exhibits an upward trend. Under the same transmit power gain, the algorithm employed in this paper demonstrates a more significant enhancement in the system sum-rate for CoMP users. By validating the RIS’s role in signal enhancement, applying the algorithm in a CoMP-NOMA system without the assistance of RISs results in a significant reduction in the sum-rate for CoMP users due to the lack of RIS-assisted communication. This confirms that controlling RIS phase shifts can improve the communication quality for edge users. In comparison experiments where intra-group power is equally allocated, disregarding the bias introduced by the phase-shift control, due to superior channel conditions for NOMA users within the group compared to CoMP users, equal power allocation within the group violates the fairness principle and fails to maximize the communication quality for CoMP users. Thus, the jointly optimized intra-group power allocation and phase shift proposed in this paper demonstrate both rationality and effectiveness in enhancing the communication quality for system edge users.

**Figure 5:**
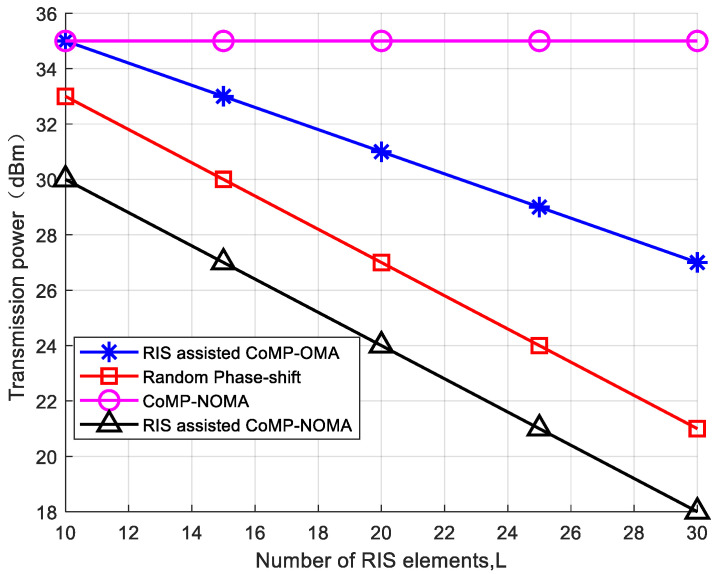
The relationship between transmission power and reflecting units.

To verify the impact of the number of reflecting units on the system’s transmit power, with a fixed number of users K = 24, as shown in Figure 5, as the number of reflection units increases, the available phase adjustment schemes also increase. Moderately increasing the number of reflection units can improve the transmission quality, reduce multipath effects and signal attenuation, and thus improve the communication quality and data transmission rate. When the number of reflecting units increases, the transmit power of the RIS-assisted CoMP-NOMA network system, as adopted in this paper, decreases. While there is a decreasing trend in power consumption when phase shifts are randomly generated, the system fails to achieve better performance due to the lack of optimization in the phase shift. In the RIS-assisted CoMP-OMA system, the lower spectrum utilization results in the worst performance for this scheme. In the CoMP-NOMA system without RIS assistance, the absence of reflecting units leads to constant system power consumption. Increasing the number of reflecting units can effectively reduce the system’s power consumption.

**Figure 6:**
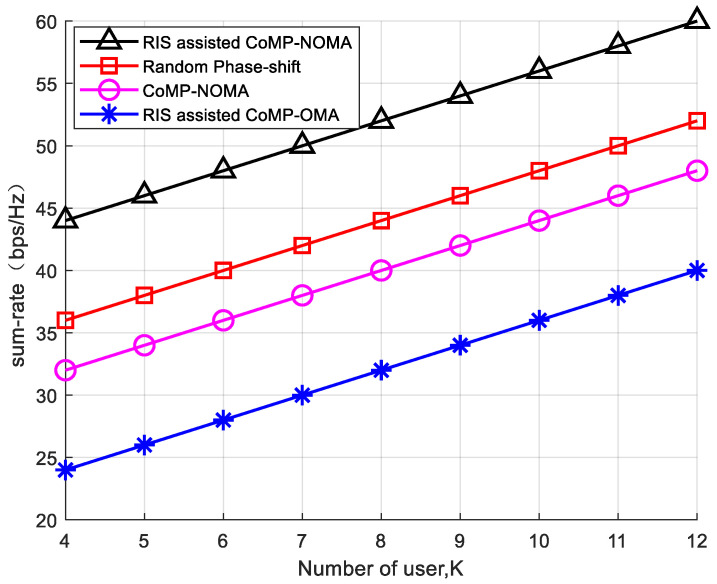
The relationship between the system rate and the users.

To verify the influence of the number of users on the system sum-rate, with a fixed number of reflecting elements L = 25 and a system transmit power of P = 30 dB, four experimental schemes were compared. As shown in Figure 6, with the increase in the number of system users, both system sum-rates exhibit an upward trend. In the RIS-assisted CoMP-NOMA system design proposed in this paper, through grouping strategies and phase optimization, with a fixed transmit power, as the number of users increases, more CoMP-NOMA clusters are formed, leading to a significant improvement in the system sum-rate.

**Figure 7:**
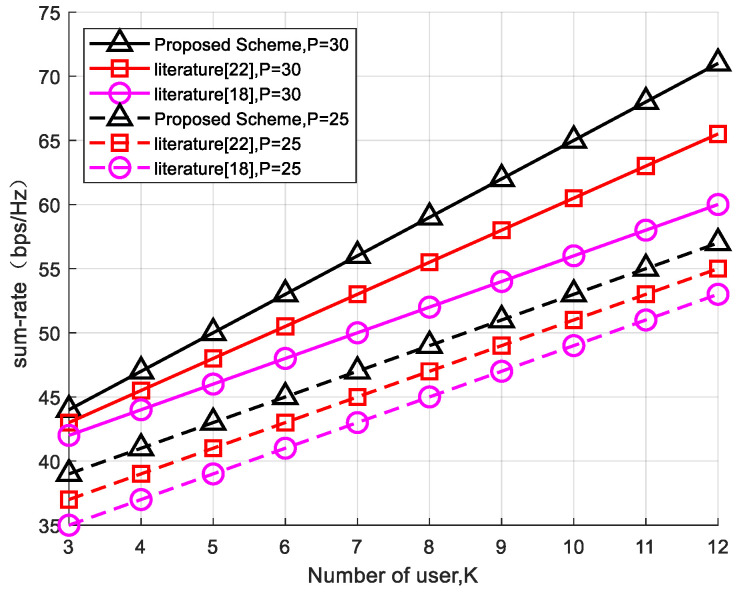
Comparison of experimental studies in the literature Mohamed, E., 2022 [22], Wang, H., 2022 [18] on changes in the system sum-rate with an increasing number of users at different powers.

The comparison between the algorithm proposed in this article and the existing technology [18,22] shows that the number of fixed reflection units is L = 25. As the number of users increases, the sum-rate of the system acts as shown in Figure 7 when the transmission power is P = 25 and P = 30. With the assistance of multiple intelligent reflection surfaces for communication, the algorithm in this article combines two user clusters, which greatly improves the sum-rate of the system from two levels of service fairness and communication quality improvement, as well as the reasonable allocation of inter-group power and intra-group power. The technology mentioned in reference Mohamed, E 2022 [22] is mainly applied in systems with single-RIS-assisted communication. However, in actual communication scenarios, with a large number of users, the resource utilization rate of single-RIS-assisted communication is relatively low. The technology proposed in reference Wang, H 2022 [18] reduces the transmission power of the system from the perspective of eliminating interference. As the number of system users increases, the energy efficiency loss caused by eliminating interference also deepens. Therefore, the system’s sum-rate is lower than that in the other two comparison schemes.

## 6. Conclusions

This article focuses on the system model of distributed RIS-assisted CoMP-NOMA systems in the downlink. It analyzes the interaction mechanisms among RIS signal enhancement, NOMA user scheduling, and multi-point cooperative transmission, as well as the factors influencing communication performance. Firstly, it establishes RIS-enhanced edge user grouping to obtain the optimal RIS-enhanced signal and enhance service fairness, then coordinates NOMA user clusters based on this foundation. Secondly, by jointly optimizing inter-group power allocation and coordinated NOMA user cluster design, the system achieves a maximized sum-rate. After obtaining the optimal solution for inter-group power allocation, intra-group power is fixed, and through alternate optimization of the intra-group power allocation and phase shifts, the system maximizes the sum-rate for CoMP users, thereby effectively improving the communication quality for edge users. Additionally, simulation experiments validate the communication performance of distributed RIS-enhanced edge user groups and the reduced user power consumption under coordinated NOMA clusters, significantly enhancing both the system sum-rate and the communication quality for edge users. Given that the premise of this study assumes that all users are statically deployed, to align with the dynamic nature of mobile communications, future research will consider dynamic user deployment to further enhance system performance.

## Figures and Tables

**Table 1:** Comparison of the proposed work with existing RIS-enabled multi-cell works.

Ref.	Cell(s)	RIS(s)	Work Objective	Beamforming	Cluster Strategy	Coordinate Cluster Strategy
[12]	Single	Single	Evaluating an IRS-enhanced mmWave-NOMAsystem	√	√	——
[15]	Single	Single	Investigating the fundamental capacity limits ofIRS-assisted multi-user wireless communications	√	——	——
[18]	Double	Single	Minimizing the transmission power	√	√	——
[22]	Double	Single	Maximizing the network sum-rate	√	——	√
[25]	Multi	Single	Maximizing the network sum-rate	√	√	——
[26]	Double	Double	Investigating outage performance	√	√	——
[27]	Multi	——	Maximizing the network sum-rate	——	√	√
[28]	Multi	Single	Minimizing the total power consumption	√	——	——
[29]	Multi	Multi	Maximizing the total spectral efficiency	√	——	——
This work	Multi	Multi	Maximizing the network sum-rate and minimizing the total power consumption	√	√	√

**Table 2:** Simulation parameter settings.

Parameter	Value
Bandwidth	10 MHz
Noise power spectral density	−105 dB
Number of users	36
Maximum number of Iterations	20
Free space loss model	125 + 36.6log10(d)
Convergence threshold	10^−3^
Minimum SINR	−10 dB
Number of reflective surfaces	3
Threshold ς	0.001~0.01
The number of reflecting elements	10~30
